# Role of ZNF143 and Its Association with Gene Expression Patterns, Noncoding Mutations, and the Immune System in Human Breast Cancer

**DOI:** 10.3390/life13010027

**Published:** 2022-12-22

**Authors:** Salma Saddeek, Rehab Almassabi, Mohammad Mobashir

**Affiliations:** 1 Department of Chemistry, Faculty of Sciences, Universty of Hafr Al Batin, Hafr Al Batin 39524, Saudi Arabia; 2 Department of Biochemistry, Faculty of Sciences, University of Tabuk, Tabuk 47512, Saudi Arabia; 3 SciLifeLab, Department of Oncology and Pathology, Karolinska Institutet, P.O. Box 1031, 17121 Stockholm, Sweden; 4 Department of Microbiology, Tumor and Cell Biology, Karolinska Institutet, Solnavägen 9, 17165 Solna, Sweden; 5 Special Infectious Agents Unit—BSL3, King Fahd Medical Research Centre, King Abdulaziz University, Jeddah 21362, Saudi Arabia

**Keywords:** ZNF143 protein, promoters, gene expression patterns, TF binding site, (non-)coding mutational profiling

## Abstract

The function of noncoding sequence variations at ZNF143 binding sites in breast cancer cells is currently not well understood. Distal elements and promoters, also known as cis-regulatory elements, control the expression of genes. They may be identified by functional genomic techniques and sequence conservation, and they frequently show cell- and tissue-type specificity. The creation, destruction, or modulation of TF binding and function may be influenced by genetic modifications at TF binding sites that affect the binding affinity. Therefore, noncoding mutations that affect the ZNF143 binding site may be able to alter the expression of some genes in breast cancer. In order to understand the relationship among ZNF143, gene expression patterns, and noncoding mutations, we adopted an integrative strategy in this study and paid close attention to putative immunological signaling pathways. The immune system-related pathways ErbB, HIF1a, NF-kB, FoxO, JAK-STAT, Wnt, Notch, cell cycle, PI3K–AKT, RAP1, calcium signaling, cell junctions and adhesion, actin cytoskeleton regulation, and cancer pathways are among those that may be significant, according to the overall analysis.

## 1. Introduction

One of the most common cancers in women identified worldwide is breast cancer (BC). Over the last 40 years, BC incidence has grown, and there has been significant development in clinical practice and diagnostic methods for detection and identification. Its annual mortality rate globally is close to 450,000 fatalities, and more than 1.4 million new patients are diagnosed with BC each year [1]. Additionally, BC is the second-leading cause of cancer-related deaths in women in industrialised countries, and the death toll from BC is considerably greater in low- and middle-income countries, such as Arabic nations, where the majority of women are diagnosed with advanced illness [2,3,4].

Cancer is thought to be a multistage process where a normal cell is transformed into a malignant cell; the malignant cells may be readily distinguished, according to research on the (epi-)genomic, transcriptome, proteome, and metabolome levels. According to earlier research, practically all kinds of cancer may be defined in terms of their ability to spread through angiogenesis, metastasis, inflammation, metabolic alterations, proliferation, and apoptosis. This multistage development has been extensively discussed, examined, and improved with the use of recently developed high-throughput methods, with a particular emphasis on (epi-)genetics as well as the transcriptome and the proteome. In peripheral blood cells from individuals with severe lupus, there is a signature of genes induced by interferon [2,4,5,6,7,8,9].

Single nucleotide variations (SNV), copy number variations (CNV), loss of heterozygosity (LOH), genomic rearrangements, and rare variants are studied at the genome level; TF binding, DNA methylation, chromatin accessibility, and miRNA are studied at the epigenome level; and gene expression, lncRNA, small RNA, and alternative splicing are studiable at the transcriptome level. Moreover, understanding epigenetics and noncoding mutations in cancer is also being tried by a number of researchers [10,11,12].

In healthy cells, chromatin accessibility and epigenetic changes work together to control gene expression. This integration of different types of genomic information also helps to explain the abnormal gene expression frequently seen in cancerous cells. Large-scale chromatin looping is involved in the repression or stimulation of gene expression. It is generally accepted that the establishment of chromatin looping, which in turn facilitates the contacts between promoters and enhancers, initiates chromatin interactions. If the genome were linear, however, it would be impossible for these interactions to arise. The precise mechanism by which chromatin connections begin has not yet been established. Recent theories, however, contend that chromatin connections are mediated by the CCCTC binding factor (CTCF)/cohesin proteins, which are crucial for direct contact between promoters and enhancers, which in turn begin and control gene expression [1,2,3,4,5].

Additionally, it is understood that ZNF143 is essential for healthy tissue growth and is present in the majority of cancer cells [13,14]. The function of noncoding sequence variations at ZNF143 binding sites in BC cells is currently not well understood. Distal elements and promoters, together known as cis-regulatory elements, control the expression of genes. They may be identified by functional genomic techniques and sequence conservation, and they frequently show cell- and tissue-type specificity. The creation, destruction, or modulation of TF binding and function can be caused by genetic modifications at TF binding sites that modify binding affinity. Therefore, noncoding mutations that target the ZNF143 binding site may be able to alter the expression of some genes in breast cancer.

Around 97% of the genome is noncoding, and the sequence variants are identified in cis-regulatory regions, the majority of which are thought to be functional genomic regions. Cis-regulatory regions include promoters and distal elements (enhancers, silencers, and insulators) that regulate and initiate gene expression and play roles in structuring and building up the higher level of chromatin organisations. Application of the next generation sequencing approach has helped in several research areas and has also helped to reveal the foundation of genetic and epigenetic underlying diseases and to refine clinical diagnosis and treatment. BC is considered a heterogeneous disease and thus has different clinical behaviours and drug responses. A research study [1,2] analysed whole genome sequences of 560 BC samples (366 samples were ER+ (65%), and 194 samples were ER- (35%)) and found that most of the driver mutations in BC were found in 93 protein coding cancer genes.

Mutations at noncoding regions such as point mutations and complex genomic rearrangements can disrupt or create new TF binding sites or affect noncoding RNA loci. Mapping of genome-wide ER binding in BC has been determined using ChIP-seq technology, and the heterogeneity of the ER binding was used as a classifier of BC ER subtypes, either positive or negative. Here, the major goal of the study in breast cancer was to investigate ZNF143 associated genes and pathways, gene expression profiling in breast cancer, and enhancers in the breast cancer cell line [1,2,3,4,5,6,7,8,9].

In this study, we have used an integrated approach to explore the relevance of ZNF143 in human breast cancer and have analysed the gene expression patterns and enhancers in human breast cancer; furthermore, we have also analysed their respective functions.

## 2. Materials and Methods

In the first phase, we used the protein–protein interaction (PPI) network database FunCoup [15] to map out all the genes connected to ZNF143. In addition, we also mapped the estimated KEGG pathways [16] and presented them as a combined network of genes and pathways. After that, GO keywords, pathways, and protein categorization were all accomplished using the PantherDB [17,18,19,20,21,22,23,24,25]. The list of ZNF143-associated genes was processed as an input gene list for PantherDB and then we executed the options for predicting the enriched GO terms, Panther pathways, and enriched protein classes.

GSE27463, GSE41324 [26], GSE43836 (https://www.ncbi.nlm.nih.gov/geo/query/acc.cgi?acc=GSE43836 (accessed on 15 October 2022)), GSE62228 (https://www.ncbi.nlm.nih.gov/geo/query/acc.cgi?acc=GSE62228 (accessed on 15 October 2022)), and GSE71898 (https://www.ncbi.nlm.nih.gov/geo/query/acc.cgi?acc=GSE71898 (accessed on 15 October 2022)) were utilized for gene expression analysis, and for differential gene expression analysis, we compared tumour samples with normal samples of the relevant samples to produce four DEGs lists. The enhancer datasets for MCF7 and MCF10A were obtained by using the HACER database [27].

The main procedures for the entire study are, in short, the processing of raw files, intensity calculations, and normalization. The three methods for normalization [28,29,30,30,31,32,33,34,35] that are most frequently used are GCRMA [36], RMA, and EB. Here, we have normalized the raw intensity using EB. After normalization, we move forward with our objective, which is to understand the patterns of gene expression and their presumed roles. MATLAB tools, such as mattest, have been utilized for statistical analysis and differential gene expression prediction. We used the Panther database for pathway analysis and created our own code for network analysis. FunCoup2.0 has been used throughout the entire investigation to generate DEG networks, and Cytoscape has been utilized to display the networks. MATLAB has been used for the majority of our code and calculations. Protein complexes, protein–protein physical interactions, and metabolic and signaling pathways are only a few examples of the four different groups of functional coupling or linkages predicted by FunCoup. Finally, it was applied to understand the gene expression patterns [37,38], and we calculated its inferred functions [17,38]. For pathway analysis, we used the KEGG [16] database and designed our own code for pathway and network analysis. The individual lists of genes (DEGs or enhancers) were prepared in text files and processed for comparative analysis as well as for KEGG pathway enrichment analysis. For Venn diagram plotting, molbiotools (https://molbiotools.com/listcompare.php (accessed on 15 October 2022)) was used, which gives the plot for the Venn diagrams and the pairwise plots and also gives the list of genes or pathways.

For generating the DEG network, FunCoup2.0 was used for all the networks throughout the work, and Cytoscape [39] has been used for network visualization. After generating the list of DEGs, the connectivities between these genes are fetched from the FunCoup2.0 network database. Once the connectivities are fetched, we then import the file with genes and connectivities into Cytoscape, where the nodes’ and edges’ color and styles are selected as per our interest. As metioned above, MATLAB was used for coding purpose, FunCoup to predict PPI networks, and Cytoscape for visualization and for more details these references could also be seen [20,24,25,40,41,42,43,44].

## 3. Results

### 3.1. Potential ZNF143 Interactors and the Biological Functions

**Figure 1:**
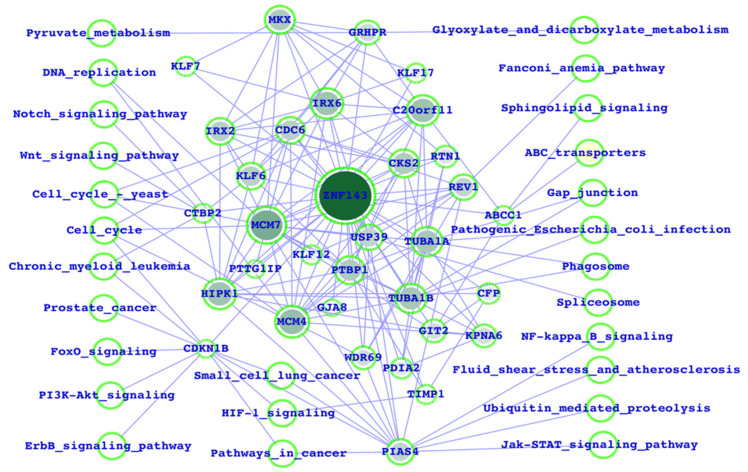
ZNF143 interactors mapped out from the PPI network database with the intra-connectivities.

Initially, we performed the mapping of ZNF143 interactors by using the PPI network database and fetched the KEGG pathways (from the KEGG pathway database) for all the interactors, including ZNF143. ZNF143, the interactors, and inferred pathways were merged together as an integrated network, which directly reflected the respective pathways (Figure 1). Furthermore, we have also mapped out the intra-connectivities between all the interactors for a better understanding of the impact of one interactor over the others. Here, we observe that MCM7/4, PIAS4, TUBA1A, C20orf11, HIPK1, and CDKN1B were among the highly connected proteins (interactors). In terms of functions, cell cycle, FoxO, PI3K-Akt, JAK-STAT, NF-kB, ubiquitin, Wnt, notch, and cancer signaling were potentially associated, and the majority of the pathways are well known to be associated with breast cancer. PIAS4, CDKN1B, and MCM7/4 directly connect to most of the inferred critical functions (Figure 1). The color-filled nodes represent the proteins/genes, and the empty nodes represent the pathways. CDKN1B, PIAS4, CTBP2, and ABCC1 are connected, with the maximum number of biological pathways with respect to the other proteins. Based on this result, we could conclude that CDKN1B, PIAS4, CTBP2, and ABCC1 could be the potential source for ZNF143 (because these genes link more biological functions) to alter the major cancer signaling pathways.

### 3.2. Functional Analysis Using Panther Database

For depth analysis of ZNF143 and the associated proteins, we performed a function analysis by using the Panther database where the GO terms, pathways, and the protein classes were analyzed. In the case of the molecular function GO term, binding, catalytic activity, and transcription regulators were predominantly present (based on percentage present in the pie chart). Among biological processes, biological regulation, cellular process, metabolic process, and signaling were dominant. In the case of the cellular component, the cellular anatomical entity was dominant, followed by the protein-containing complex. With respect to Panther pathways, the gonadotrophin releasing hormone receptor pathway was more dominant, while IFN-γ, IL signaling, JAK/STAT signaling, and Wnt signaling appear in equal percentages. These pathways are globally considered to be potentially related to cancer, including breast cancer.

**Figure 2:**
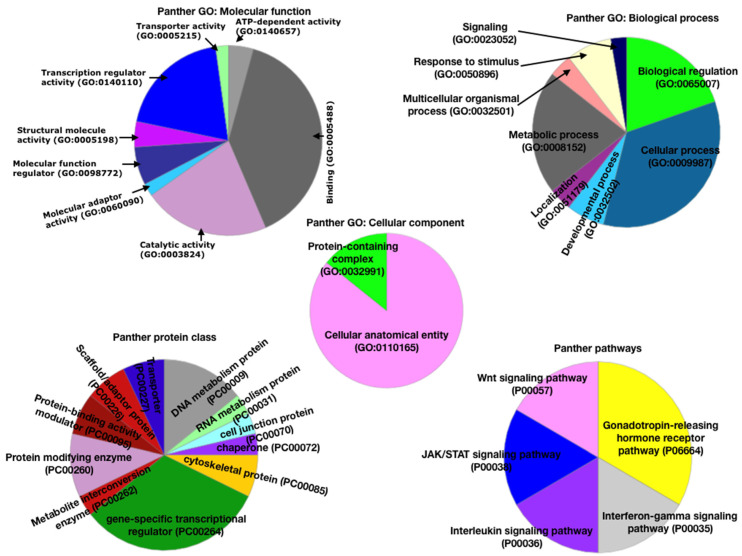
Functional classification of ZNF143 interactors mapped out from the PPI network database by using the Panther database.

In the case of protein classification, the gene-specific transcriptional regulator class appears highly dominant. The protein modifying enzyme, DNA metabolism protein, protein-binding activity modulator, scaffold/adaptor protein, transporter, and cytoskeletal protein were present in closely related percentages. There were other protein classes also present (Figure 2). The above-mentioned protein classes are those that have the proteins and are known to be associated with a large number of human diseases, including all types of cancer. Thus, we could say that these enriched protein classes are associated with the selected cancer type, i.e., breast cancer.

### 3.3. Gene Expression Patterns in Human Breast Cancer and the Analysis of Enhancers in the Breast Cancer Cell Line

**Figure 3:**
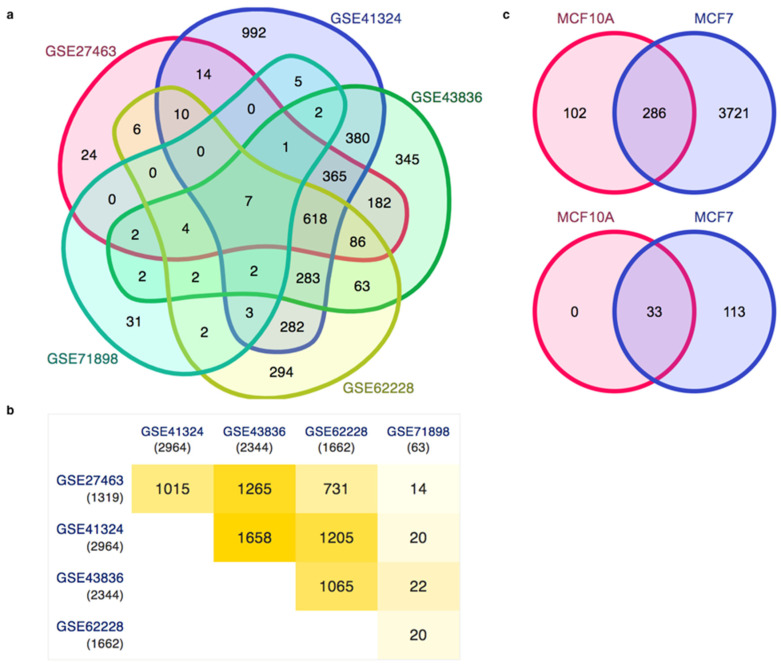
(**a**,**b**) Comparative analysis of the gene expression patterns in breast cancer and (**c**) the enhancers (HACER) for MCF10A (normal breast cell line) and MCF7 (breast cancer cell line).

Further, we mapped out the gene expression profiling for the human breast cancer cell line MCF7 and compared them altogether. We observe that GSE27463 shares 1015 genes with GSE41324, 1265 genes with GSE43836, 731 genes with GSE62228, and 14 genes with GSE71898, as shown in Figure 3a,b. After analyzing the gene expression profiling, we have used the enhancer datasets for the breast cancer cell line MCF7 and the healthy breast cell line MCF10A from HACER, and we compared the closest genes and the enriched pathways for the closest genes. From the enhancer analysis, it appears that there are 286 genes in common between MCF7 and MCF10A. There were 3721 genes specific to MCF7, while 102 genes were specific to MCF10A, which means that there is a large number of enhancers which are specific to breast cancer cell lines; this is also the case in terms of functional analysis (Figure 3c).

**Figure 4:**
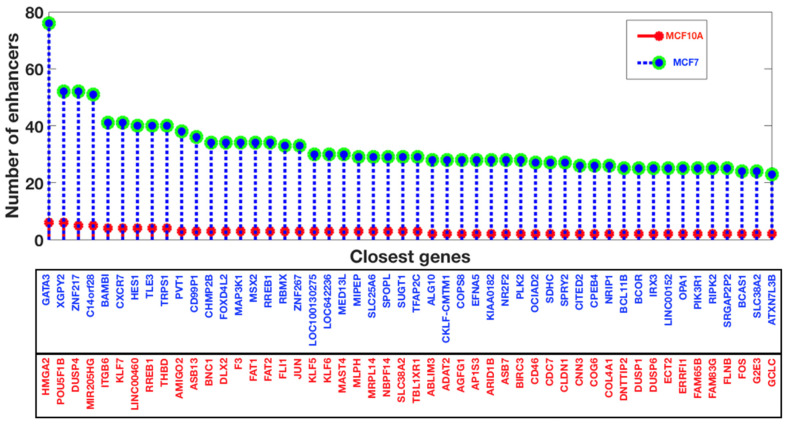
Top 50 genes in terms of enhancers (HACER) for MCF10A (normal breast cell line) and MCF7 (breast cancer cell line).

To explore it further, the top 50 genes were analyzed and plotted (Figure 4), where we show the number of enhancers in the cases of top-ranked genes. The MCF10A top 50 genes were also plotted, where we observed that the number of enhancers was comparatively very low in the control cell line compared to the breast cancer cell line.

**Figure 5:**
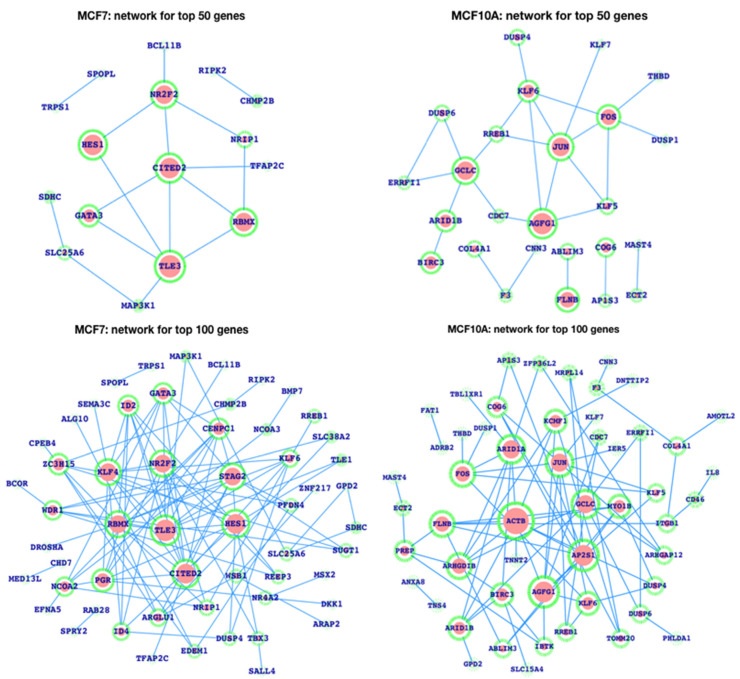
Network for top 100 and 50 genes in terms of enhancers (HACER) for MCF10A (normal breast cell line) and MCF7 (breast cancer cell line).

**Figure 6:**
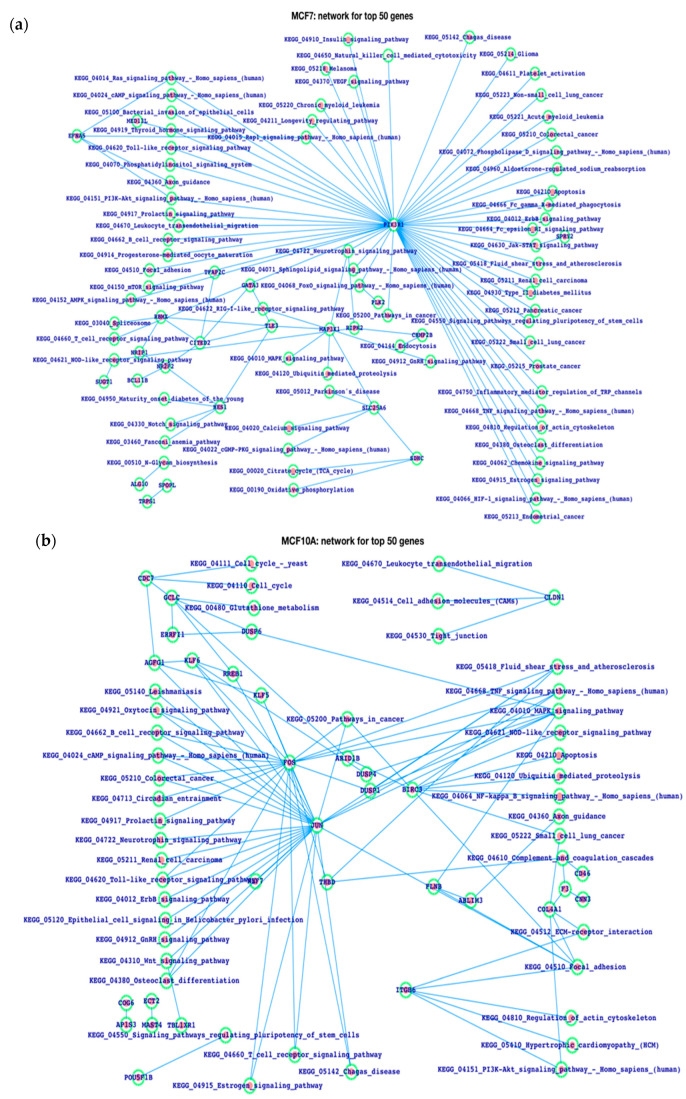
The (**a**) MCF7 and (**b**) MCF10A networks. The top 50 genes were analyzed and plotted to show the associated pathways and the mode of connectivity.

After plotting the top ranked genes (50 and 100) of MCF7 and MCF10A, the network for the respective genes weasalso plotted, wherein we observed that the MCF7 network is densely connected with respect to MCF10A for the top 100 genes, while the connectivity is close to each other for the top 50 genes (Figure 5). Moreover, we have also plotted the network of genes and the pathways together for the top 50 genes’ network (Figure 6), in which we observe the most critical and more numerous pathways are associated with the MCF7 network compared to the MCF10A network. We have explored the networks of both MCF7 and MCF10A, and, in the case of the MCF7 network, PIK3R1 is the gene that controls the maximum number of biological functions. There is a large number of pathways which are potentially associated with breast cancer, such as apoptosis, ErbB, JAK-STAT, HIF-1, regulation of actin cytoskeleton, TNF, pathways in cancer, TCR, BCR, TLR signaling pathways, etc. In the case of the MCF10A network, JUN and FOS proteins infer a large number of critical signaling pathways but many fewer than PIK3R1 of MCF7, and there were less dense protein pathway connections than in MCF7. Thus, MCF7 and MCF10A networks are very different.

## 4. Discussion

As previously stated, we started by focusing on the genes that were specifically connected to ZNF143 and their intra-associations within the framework of a network-based public network database, for which the FunCoup network database was used. The predicted pathways for each of these genes have been mapped using the KEGG pathway database. These essential facts lead us to the conclusion that the majority of the genes associated with ZNF143 are linked to the most significant cancer-related pathways. We are motivated to thoroughly study each of the aforementioned pathways’ roles in breast cancer because the majority of them are well-established and acknowledged to either directly or indirectly regulate numerous types of human cancer. Here, CDKN1B, PIAS4, CTBP2, and ABCC1 could be the potential source for ZNF143 to alter the major cancer signaling pathways.

Furthermore, we have displayed the co-expressed genes for ZNF143 as well as the pathways directly connected to ZNF143 in cases of breast cancer. The cell cycle, cancer, ErbB, HIF1a, NF-kB, Foxo, JAK–STAT, Wnt, and Notch pathways are among the main pathways connected to ZNF143 or its interactors, and these pathways are well-known to be potentially associated with breast cancer [32,34,45,46,47,48,49,50,51,52,53]. Thus, it may suggest that ZNF143 could be one of the most significant genes that may control the breast cancer-related signaling pathways. There are a number of works where the research has concentrated on ZNF143; however, our work is distinctive in that it gives fundamentals related to ZNF143 in a straightforward manner [44,45,46,47,48]. Most biological functions in the MCF7 network are controlled by the PIK3R1 gene, and many other pathways, including apoptosis, ErbB, JAK–STAT, HIF-1, the control of the actin cytoskeleton, TNF, cancer-related pathways, TCR, BCR, and TLR signaling pathways, may also be related to breast cancer. JUN and FOS proteins infer a high number of crucial signaling pathways in the MCF10A network, but considerably less than PIK3R1 does in MCF7. There were fewer dense protein pathway connections than in MCF7. The MCF7 and MCF10A networks are therefore very different from one another.

It is widely known that at the anchor regions of chromatin loops, CTCF–cohesin complex and transcription factor ZNF143 often co-bind. Researchers have also conducted a genome-wide experiment to examine ZNF143’s functional roles in chromatin loops, in which they used computational and experimental methods to look at how ZNF143 influences chromatin loop regulation. The underlying ZNF143-binding sites, ZNF143–CTCF co-binding sites, and ZNF143–CTCF–RAD21 co-binding sites have been identified through the combined study of the ZNF143 and CTCF motifs. Their findings demonstrate that the ZNF143–CTCF–RAD21 co-binding sites are enriched with CTCF motifs but depleted of ZNF143 motifs, indicating that ZNF143 may act as a cofactor rather than the pioneer factor of the ZNF143–CTCF–cohesin complex when it comes to direct genome binding of CTCF but not ZNF143 [54,55,56]. They carried out an siRNA experiment to reduce ZNF143’s expression level in the HEK293T cell line, and then they performed in situ Hi–C on the negative control and ZNF143-silenced HEK293T cells to investigate the regulatory impact of ZNF143 on chromatin loops. Comparative analysis reveals that in the ZNF143-silenced HEK293T cells, the majority of chromatin loops are destroyed or at the very least attenuated. The intricate roles played by ZNF143’s decrease in controlling chromatin loops are indicated by the limited percentage of chromatin loops that are either strengthened or acquired. They used aggregate peak analysis to look at the chromatin loop differences between negative control and ZNF143-silenced cells in order to further validate the loop analyses. The analysis demonstrates that after ZNF143 silencing, loop strength changes occur in both the lost and gained chromatin loops. Overall, their research demonstrates that ZNF143 can control chromatin loops by functioning as a cofactor of the CTCF–cohesin complex, and that ZNF143 expression is primarily eliminated or destabilized when ZNF143 is knocked down [54,55,56,57,58].

A cellular mechanism called autophagy removes and utilizes extraneous or broken parts to maintain cellular homeostasis. Importantly, a prior study discovered that breast cancer cells with low levels of ZNF143 expression outlived control cells (MCF7 sh-Control) under starvation when the autophagy-inhibiting drug chloroquine was present. Additionally, MCF7 sh-ZNF143 cells had more autophagic vesicles than MCF7 sh-Control cells did, and cells with less ZNF143 had changes in the autophagic process-related proteins Beclin1, p62, and ATGs. The stability of p53 was impacted by ZNF143 knockdown, which revealed MG132’s dependence on the proteasome inhibitor. NAD(P)H quinone dehydrogenase 1 (NQO1) may be important for the stability of p53, according to data from proteome profiling in breast cancer cells with reduced ZNF143. Together, they demonstrate that a fraction of breast cancer cells with reduced ZNF143 expression may show improved survival through an autophagic process by controlling the p53-Beclin1 axis, validating the requirement of limiting autophagy for the most effective therapy [13,59,60,61,62].

Additionally, earlier research has indicated that ZNF143 influences the motility of colon cancer cells. In breast cancer, ZNF143 was further described. ZNF143 expression in healthy tissues and tissues from different stages of metastatic breast cancer was examined using immunohistochemistry. Notably, ZNF143 was differentially expressed in the ductal and glandular epithelia of healthy breast tissues, and this expression was reduced as the tissue progressed toward malignancy. Short-hairpin (sh) RNA-lentiviral particles against ZNF143 were used to infect benign breast cancer cells in order to knock down ZNF143 and learn more about the molecular mechanism underlying how it influences breast cancer progression (MCF7 sh-ZNF143). When compared to MCF7 sh-Control cells, MCF7 sh-ZNF143 cells displayed distinct cell–cell interactions and actin filament (F-actin) architectures. Breast cancer cells with ZNF143 knockdown exhibited greater cellular motility in migration and invasion experiments. The restoration of ZNF143 expression decreased this. These findings suggest that ZNF143 expression aids in the development of breast cancer [13,63].

We have concentrated on ZNF143 and its effect on human breast cancer, as mentioned above in the discussion section. In addition, we have further analyzed MCF7 and MCF10A in terms of enhancers, and their networks have also been plotted and examined. An improved understanding of ZNF143 in the context of human breast cancer as well as enhancers in breast cancer and normal breast cell lines results from the integrated approach.

## 5. Conclusions

We draw the conclusion from this study that the transcription factor ZNF143 may be essential since it seems that ZNF143 regulates a broader variety of biological processes and pathways linked to breast cancer. Highly significant genes include MCM7, MCM4, PIAS4, TUBA1A, C20orf11, HIPK1, and CDKN1B. Function-wise, the majority of the pathways are known to be linked to breast cancer, including the cell cycle, FoxO, PI3K-Akt, JAK-STAT, NF-kB, ubiquitin, Wnt, notch, and cancer signaling. Most of the inferred key functions are directly connected via PIAS4, CDKN1B, and MCM7/4. Proteins and genes are represented by color-filled nodes, while pathways are represented by empty nodes. Compared to the other proteins, CDKN1B, PIAS4, CTBP2, and ABCC1 are linked to the most biological pathways. Based on this finding, we might hypothesize that ZNF143’s potential to change the main cancer signaling pathways may originate from CDKN1B, PIAS4, CTBP2, and ABCC1.

## Data Availability

Not applicable.
